# Sarcopenia as a predictor of negative health outcomes in patients with type 2 diabetes mellitus: a systematic review and meta-analysis

**DOI:** 10.1186/s13098-025-01998-w

**Published:** 2025-11-05

**Authors:** Hao Bai, Yaqing Liu, Longhan Zhang, Lingqiao Song, Yiting Pan, Zeyuan Long, Li Liao

**Affiliations:** School of Nursing, University of South China, Hengyang, Hunan China

**Keywords:** Type 2 diabetes, Sarcopenia, Mortality, Cardiovascular diseases, Systematic review

## Abstract

**Background:**

Sarcopenia represents a considerable public health issue and is associated with increased mortality and complication rates in patients with type 2 diabetes mellitus (T2DM). Existing evidence regarding adverse outcomes in patients with T2DM and sarcopenia is currently scattered and limited, and comprehensive evidence is lacking.

**Methods:**

A comprehensive search of Embase, PubMed, Scopus, and Web of Science was conducted to identify relevant studies that assessed the impact of sarcopenia on mortality, cardiovascular disease (CVD), and complications in individuals with T2DM. The quality of the included studies was evaluated using the Newcastle-Ottawa Scale and the Joanna Briggs Institute Critical Appraisal tool. The pooled hazard ratios and odds ratios, along with their corresponding 95% confidence intervals for mortality, CVD, and complication estimates, were analyzed.

**Results:**

Fifteen studies were included in the meta-analysis. The overall risk of bias across the studies was low. Patients with T2DM and sarcopenia had a considerably elevated risk of mortality, with a pooled hazard ratio of 1.72 (95% CI = 1.28–2.32). Similarly, sarcopenia was associated with an increased hazard ratio for CVD of 1.94 (95% CI = 1.67–2.25). Furthermore, sarcopenia was associated with an elevated risk of developing diabetic complications, as evidenced by a hazard ratio of 1.12 (95% CI = 1.09–1.15) and an odds ratio of 2.49 (95% CI = 1.53–4.05).

**Conclusions:**

Sarcopenia is predictive of adverse outcomes, including mortality, CVD, and diabetic complications, in patients with T2DM. Our findings underscore the clinical imperative for integrating sarcopenia assessment into routine T2DM management to facilitate early risk stratification.

**Supplementary Information:**

The online version contains supplementary material available at 10.1186/s13098-025-01998-w.

## Introduction

Type 2 diabetes mellitus (T2DM) has emerged as a major global health challenge, affecting nearly 500 million individuals worldwide and exhibiting a continuously increasing prevalence [1]. It is characterized by insulin resistance and is often accompanied by obesity [2]. Insulin resistance in T2DM is part of a broader multisystemic inflammatory disorder which, together with genetic and environmental contributors, predisposes to cardiovascular and other complications [3–5]. Among the various complications of T2DM, sarcopenia has recently garnered significant attention as both a complication and an independent predictor of detrimental health outcomes in the diabetic population [6–8].

Sarcopenia—a geriatric syndrome characterized by progressive loss of muscle mass and diminished muscle function [9]—afflicts approximately 15–20% of T2DM patients [10]. This high prevalence results from complex interactions among metabolic dysregulation, chronic inflammation, and systemic dysfunction [11–13]. In particular, systemic hyperinflammation plays a central role in these interactions [14]. Consistent with its emerging role in T2DM as both complication and predictor, sarcopenia contributes to multiple adverse outcomes, including increased mortality, disability, and fall susceptibility [15, 16], profoundly compromising patients’ quality of life and generating significant socioeconomic burdens [17].

Given that T2DM accelerates the progression of muscle atrophy and functional decline [18], the coexistence of sarcopenia substantially complicates clinical management and amplifies risks of serious comorbidities [19, 20]. Despite growing evidence linking sarcopenia to deleterious health sequelae in T2DM patients [21–23], the literature lacks a systematic synthesis to clarify its prognostic significance. Thus, this study aimed to elucidate the impact of sarcopenia on the prognosis of T2DM patients and to provide evidence to support routine sarcopenia assessment in this population.

## Materials and methods

The review protocol was registered in the PROSPERO database. The PROSPERO registration number is CRD420251004812. The study was performed according to the Preferred Reporting Items for Systematic Reviews and Meta-analyses (PRISMA) guidelines [24], which are shown in Supplementary Table 4.

### Search strategy

We performed a systematic literature search for relevant articles restricted to English-language publications, and an electronic search was performed in the following databases: Embase, PubMed, Scopus, and Web of Science up to March 12, 2025. Manual reference screening of included studies and relevant reviews was performed to identify additional eligible records. The search details are shown in Supplementary Table 1.

### Eligibility criteria

The inclusion criteria were as follows: (1) study design: observational studies, including cross-sectional studies as well as prospective and retrospective cohort studies; (2) study population: individuals aged ≥ 18 years with a clinical diagnosis of T2DM; and (3) exposure: sarcopenia. Diagnostic parameters for sarcopenia, such as the skeletal muscle mass index (SMI) and the criteria established by the Asian Working Group on Sarcopenia (AWGS), the European Working Group on Sarcopenia in Older People (EWGSOP), and the Foundation for the National Institutes of Health (FNIH), are clearly defined in the literature. (4) Outcomes: Studies that reported data on mortality, cardiovascular events, and complications in patients with T2DM, both with and without sarcopenia, were included. If multiple studies from the same cohort reported different outcomes or distinct subpopulations, all relevant studies were included.

### Exclusion criteria

The exclusion criteria were as follows: (1) studies not published in English; (2) duplicate publications; (3) studies with outcome data that could not be extracted; and (4) review articles, letters, editorials, conference abstracts, and commentaries.

### Data extraction and quality assessment

Two independent reviewers (HB and YL) screened all titles and abstracts to identify potentially eligible studies. If a study appeared to meet the inclusion criteria based on the abstract, the full text was retrieved and assessed to confirm eligibility. Data extraction was conducted only after the final inclusion of studies following full-text review. Data extraction was performed independently by two reviewers (HB and YL) using a standardized data collection form. The following information was extracted from each study: first author, year of publication, study design, study region, sample characteristics, mean age of the participants, definition of sarcopenia, method of muscle mass assessment, and outcomes. Additionally, hazard ratios (HRs) and odds ratios (ORs), adjusted for confounding factors, were calculated for each study. Any disagreements encountered during study selection or data extraction were resolved by consensus, with a third reviewer (LL) consulted when necessary. The methodological quality of the included studies was independently assessed by two reviewers. Two reviewers assessed the quality of the included studies using the Joanna Briggs Institute Critical Appraisal tool [25] for cross-sectional studies and the Newcastle‒Ottawa Quality Scale [26] for cohort studies. The overall certainty of the evidence for each endpoint was assessed according to the Grading of Recommendations Assessment, Development and Evaluation (GRADE) approach.

### Statistical analysis

Data were presented as proportions via descriptive statistics, and statistical uncertainties were presented as 95% confidence intervals (CIs). The characteristics and key findings of the included studies were summarized in a narrative synthesis. A random-effects model was used to combine data from individual studies to derive an overall quantitative estimate of the effect of sarcopenia on health outcomes in individuals with T2DM. We calculated the pooled estimates, including HRs and ORs, and plotted them as forest plots. Adjusted outcome data were controlled for critical confounding variables via multivariate analyses; therefore, they were considered more reliable estimates. Due to variations in geographic distribution and outcome assessment among studies, we used a random-effects model rather than a fixed-effects model, regardless of statistical evidence for heterogeneity. Begg’s and Egger’s tests were used to detect publication bias. The p-values of Begg’s and Egger’s tests (>0.05) suggested that there was no significant publication bias. The trim-and-fill method aims to estimate potentially missing studies due to publication bias in the funnel plot and adjust the overall effect estimate [27]. We performed a sensitivity analysis via the leave-one-out approach (omitting each study individually) to examine the impact of clinical heterogeneity and methodological bias on adverse outcome estimates. All analyses were conducted using RevMan version 5.3 (The Cochrane Collaboration) and Stata (version 18.0; Stata Corp, College Station, TX, USA), with 2-sided *p* < 0.05 considered statistically significant.

This study conducted subgroup analyses to explore the potential sources of heterogeneity and obtain further information according to the following factors: definition of sarcopenia (AWGS vs. EWGSOP vs. SMI); assessment method of muscle mass (dual-energy X-ray absorptiometry (DXA) vs. bioelectrical impedance analysis (BIA) vs. computed tomography (CT)); and study region (Asia vs. other regions).

## Results

### Study selection

**Fig. 1:**
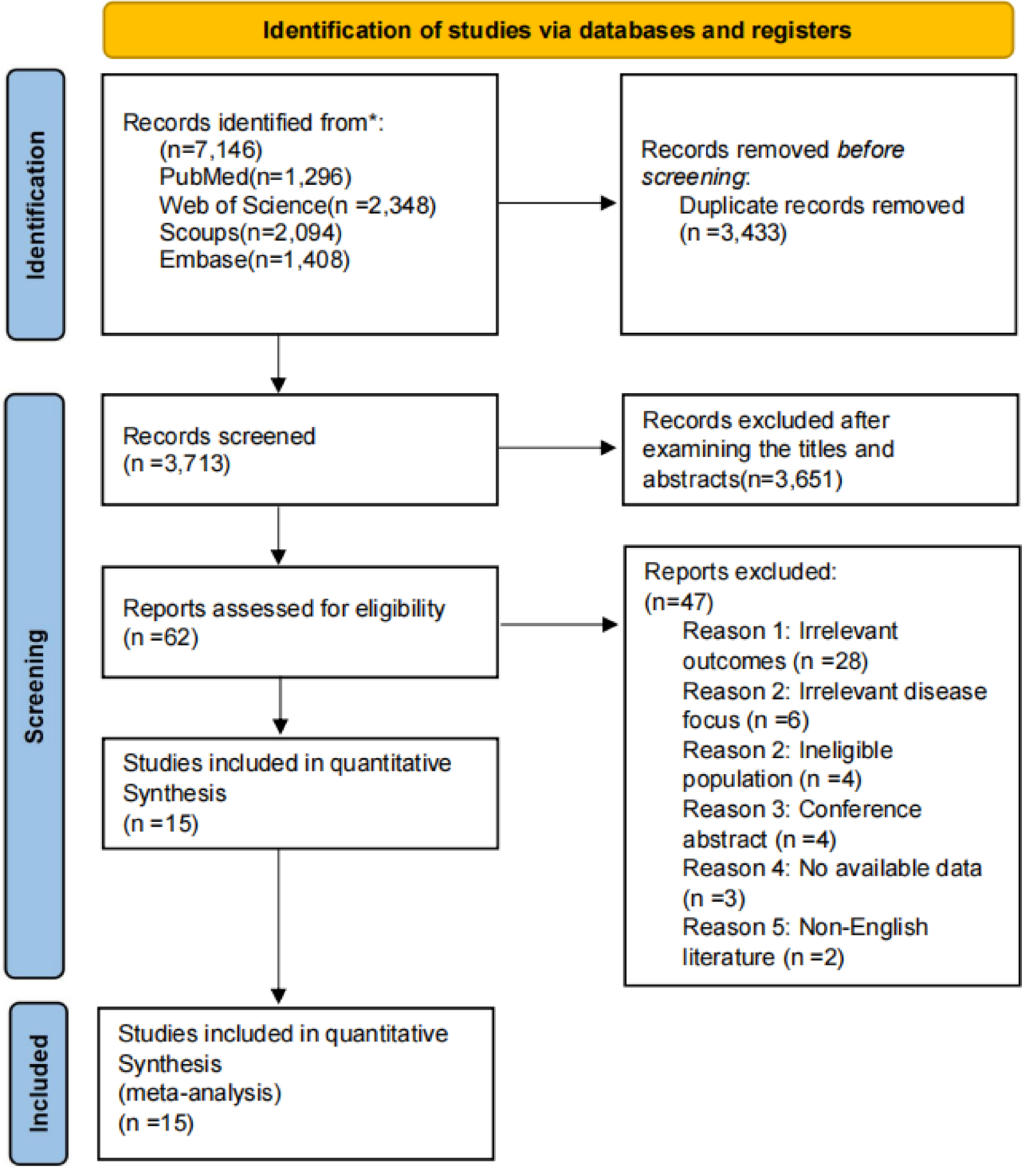
Flow diagram of the literature search and study selection process

A PRISMA flow diagram summarizing the process of selecting the studies included in this analysis is shown in Fig. 1. After removing duplicates, 3713 relevant articles were reviewed. After screening the titles and abstracts, 62 articles were considered potentially eligible. After a full-text review, 15 articles [28–42] fulfilled the entry criteria and were subjected to the final analysis. Supplementary Table 5 provides the specific reasons for excluding studies (*n* = 47).

### Study characteristics

The characteristics of the 15 included studies are presented in Table 1. The median sample size was 762 (range, 238–201698). Of these, 12 studies ([28, 29, 31, 32, 34–36, 38–42] were cohort studies, and three studies ([30, 33, 37] were cross-sectional studies. The average baseline age of the participants ranged from 21 to 82 years, and the length of follow-up ranged from 1 to 15.65 years. The proportions of males and females were 49.4% and 50.6%, respectively. The study populations were derived from both large population-based cohorts and hospital-based cohorts. The included studies were conducted in different geographic regions; most were from Asia (*n* = 12) ([28, 30–33, 36–42], Europe (*n* = 2) ([29, 34], or South America (*n* = 1) ([35]. In the 15 included studies, four different definitions of sarcopenia were adopted: SMI^[28,32,33,36–38],^ AWGS ([31, 39–42], EWGSOP ([29, 34, 35], and FNIH ([30]. Four different methods for assessing muscle mass were used, including DXA ([30, 31, 33, 37, 40–42], BIA ([29, 34, 39], CT ([28, 32, 36, 38], and calf circumference (CC) ([35].

### Assessment of bias

The quality of the included studies was moderate or reasonable, with scores ranging from six to nine points. The Newcastle‒Ottawa scale quality assessment results for the included studies are shown in Supplementary Table 2.

### Associations between sarcopenia and increased mortality in T2DM patients

**Fig. 2:**
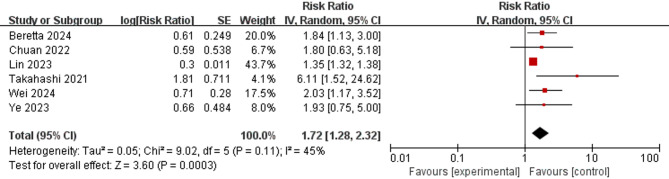
Forest plot of the association between sarcopenia and mortality

Six cohort studies reported the effects of sarcopenia on all-cause mortality in patients with T2DM (see Fig. 2). Meta-analysis revealed that patients with T2DM and sarcopenia had a considerably elevated risk of mortality, with a pooled HR of 1.72 (95% CI = 1.28–2.32; *p* < 0.001).

Subgroup analyses were conducted on the basis of the definition of sarcopenia, study region, and assessment method of muscle mass. According to the AWGS criteria [31, 39, 41], the pooled HR for mortality was 2.41 (95% CI = 1.24–4.69; *p* = 0.009). Using the EWGSOP criteria [29, 35], the pooled HR was 1.92 (95% CI = 1.34–2.77; *p* < 0.001). The SMI criteria [32] yielded a pooled HR of 1.35 (95% CI = 1.32–1.38; *p* < 0.001). Collectively, these results indicate that T2DM patients with sarcopenia face a higher mortality risk compared with those without sarcopenia (see Supplementary Fig. 1). The subgroup analysis also confirmed previous results on the basis of geographic region. The HR was 1.77 (95% CI = 1.07–2.94; *p* = 0.03) for Asian regions [31, 32, 39, 41] and 1.92 (95% CI = 1.34–2.77; *p* < 0.001) for other regions [29, 35] (see Supplementary Fig. 2). When DXA [31, 41] was used to assess muscle mass, the pooled HR was 1.88 (95% CI = 0.93–3.80; *p* = 0.08), whereas the pooled HR of BIA [29, 39] was 2.90 (95% CI = 1.06–7.96; *p* = 0.04), that of CT [32] was 1.35 (95% CI = 1.32–1.38; *p* < 0.001), and that of CC [35] was 1.72 (95% CI = 1.28–2.32; *p* = 0.01), which indicated varying risks (see Supplementary Fig. 3).

### Associations between sarcopenia and increased CVD in T2DM patients

**Fig. 3:**
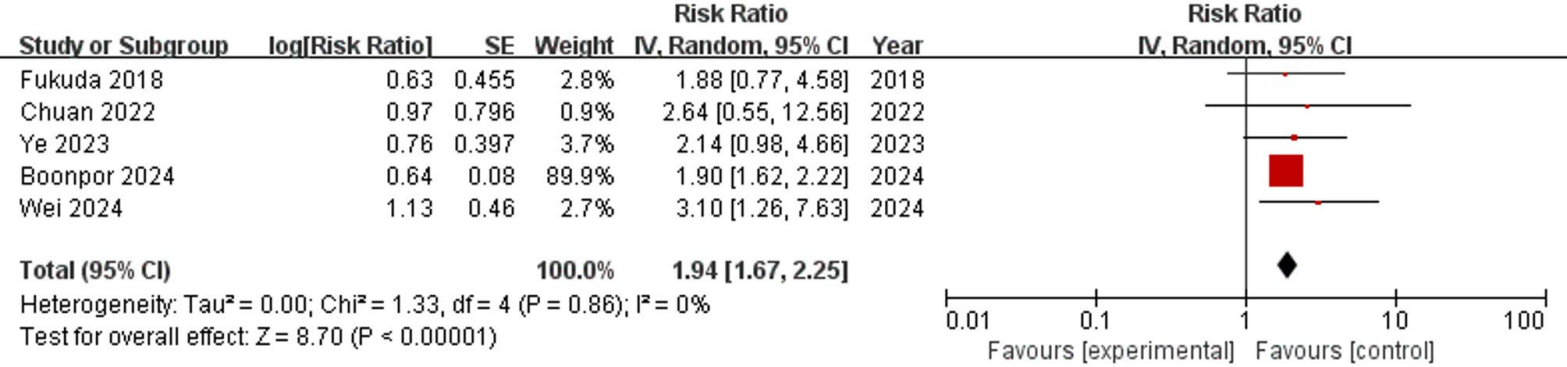
Forest plot of the association between sarcopenia and CVD

Five studies [29, 31, 34, 40, 41] reported adjusted data on the association between sarcopenia and CVD. The pooled HR of these studies was 1.94 (95% CI = 1.67–2.25; *p* < 0.001), indicating that sarcopenia was associated with an increased HR for CVD (see Fig. 3).

The definition of sarcopenia and the assessment method for muscle mass were further analyzed in subgroup analyses. Among patients with T2DM, sarcopenia assessed by both AWGS [31, 40, 41] and the EWGSOP [31, 34] demonstrated an increased risk of CVD compared to robust individuals, with pooled HRs of 2.09 (95% CI = 1.21–3.62; *p* = 0.009) and 1.97 (95% CI = 1.53–2.52; *p* < 0.001), respectively(see Supplementary Fig. 4). When DXA [31, 40, 41] was used to assess muscle mass, the pooled HR was 2.09 (95% CI = 1.21–3.62; *p* = 0.009), and that of BIA [31, 34] was 1.97 (95% CI = 1.53–2.52; *p* < 0.001), which is consistent with the results of the definition of sarcopenia (see Supplementary Fig. 5).

### Associations between sarcopenia and increased incidence of complications in T2DM patients

**Fig. 4:**
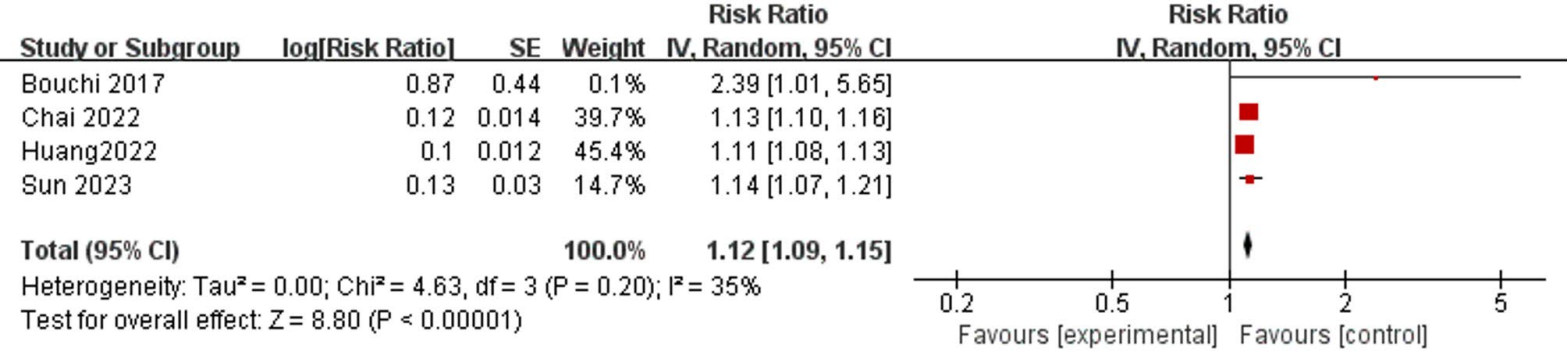
Forest plot of the associations between sarcopenia and complications

Four studies provided adjusted HR data on the associations between sarcopenia and complications, including diabetes-associated renal dysfunction [38, 42], diabetic stroke [28], and diabetes-induced dementia [36]. The pooled adjusted HR was 1.12 (95% CI = 1.09–1.15; *p* < 0.001), indicating that sarcopenia was associated with increased T2DM patient-related complications (see Fig. 4).

Three studies provided adjusted OR data on the associations between sarcopenia and complications, including diabetes-associated renal dysfunction [33, 37] and diabetic stroke [30]. With a pooled adjusted OR of 2.49 (95% CI = 1.53–4.05; *p* < 0.001), sarcopenia showed an association with a higher risk of complications (see Supplementary Fig. 6).

**Table 1:** Characteristics of the included studies

Author	Year	Region	Source of participant	Study Design	Sample (total-male‒female)	Age	Definition of Sarcopenia	Assessment method of muscle Mass	Follow-up(year)	Outcomes measure	Effect size
Chai [28]	2022	Taiwan	NHIRD	RCS	104120-56798-47322	58.04 ± 14.35	SMI	CT	6.54 ± 3.96	stroke	HR
Wei [29]	2024	UK	UK biobank	RCS	13392-7901-5500	37–73	EWGSOP	BIA	12.56 ± 3.09	mortality, CVD	HR
Park [30]	2021	Korea	The diabetes center in Wonju severance christian hospital (2017–2019)	CSS	1230-721-509	62.9 ± 10.0	FNIH	DXA	NA	CVD, stroke	OR
Ye [31]	2023	China-Mainland	First Affiliated hospital of Chongqing medical university (2015–2019)	RCS	386-177-209	≥ 60	AWGS	DXA	3.92	mortality, CVD	HR
Lin [32]	2023	Taiwan	NHIRD	RCS	201698-94015-107683	60.44 ± 14.61	SMI	CT	7.96 ± 4.62	mortality, CVD	HR
Chung [33]	2018	Korea	KSOS	CSS	409-217-192	45–77	SMI	DXA	NA	CKD, albuminuria	OR
Boonpor [34]	2024	UK	UK biobank	RCS	11974-8120-3854	40–70	EWGSOP	BIA	10.7(9.5–11.5)	CVD, stroke	HR
Beretta [35]	2024	Brazil	University hospital cohort, Brazil (2015–2017)	PCS	309-153-156	73.26 ± 6.37	EWGSOP	CC	1	morality	HR
Sun [36]	2023	Taiwan	Taiwan cancer registry database	RCS	41,294--	> 60	SMI	CT	NR	Dementia	HR
Yang [37]	2016	China-Mainland	The First Affiliated hospital of Fujian medical university (2007–2014)	CSS	762-501-261	21–82	SMI	DXA	NA	renal function	OR
Huang [38]	2022	Taiwan	NHIRD	RCS	105166-48436-56730	58.96 ± 14.56	SMI	CT	7.94 ± 4.18	diabetic nephropathy	HR
Takahashi [39]	2021	Japan	Kyoto prefectural university of medicine hospital cohort and Kameoka municipal hospital cohort	PCS	396-232-164	71.3(6.3)	AWGS	BIA	40.5	mortality, CVD	HR
Fukuda [40]	2018	Japan	Tokyo medical and dental university hospital (2008–2015)	RCS	716-380-336	65 ± 13	AWGS	DXA	2.6(2.1–3.2)	CVD	HR
Chuan [41]	2022	China-Mainland	The ageing and body composition of diabetes cohort	RCS	386-177-209	67.91 ± 6.10	AWGS	DXA	3.46 ± 1.15	mortality, CVD	HR
Bouchi [42]	2017	Japan	TMDU hospital (2012–2016)	RCS	238-145-93	64 ± 12	AWGS	DXA	2.6	albuminuria	HR

AWGS, Asian Working Group on Sarcopenia; BIA, bioelectrical impedance analysis; CT, computed tomography; CVD, cardiovascular disease; CKD, chronic kidney disease; CSS, cross-sectional study; DXA, dual-energy X-ray absorptiometry; EWGSOP, European Working Group on Sarcopenia in Older People; FNIH, Foundation for the National Institutes of Health; KSOS, The Korean Sarcopenic Obesity Study; NA, not available; NR, not reported; NHIRD, National Health Insurance Research Database; PCS, prospective cohort study; RCS, retrospective cohort study; SMI, skeletal muscle mass index; UK Biobank, United Kingdom Biobank

## Discussion

Sarcopenia adversely affects the prognosis and complicates the treatment of T2DM. It increases patient vulnerability to comorbidities and compromises resilience. Furthermore, sarcopenia is associated with poorer glycemic control, reduced tolerance to medications, and lifestyle interventions. Although its adverse effects on mortality, cardiovascular disease, and diabetes-associated complications have been reported, the clinical evidence has not yet been systematically clarified.

To the best of our knowledge, no previous study has systematically integrated evidence from disparate sources to investigate the associations of sarcopenia with adverse outcomes in T2DM patients. This study addresses this gap by providing a comprehensive synthesis of current evidence. This study included 15 eligible articles that assessed the impact of sarcopenia on T2DM. Evidence suggests that sarcopenia is a risk factor for adverse outcomes in this population. Patients with sarcopenia had an elevated risk of all-cause mortality (adjusted HR = 1.72) and CVD events (adjusted HR = 1.94) compared to those without sarcopenia. Additionally, T2DM patients with sarcopenia demonstrated a higher incidence of diabetes-related complications (adjusted HR = 1.12, adjusted OR = 2.49) relative to their non-sarcopenic counterparts. Sensitivity analyses revealed that none of the studies dominated the outcomes.

Previous meta-analyses [43, 44] have explored the prevalence and risk factors for sarcopenia in patients with T2DM, providing valuable insights. The odds of sarcopenia in T2DM patients are 1.55-fold greater than those in nondiabetic patients, driven by impaired muscle performance rather than mass alone [45, 46]. Despite the diagnostic variability, early multidisciplinary interventions are critical for mitigating adverse outcomes [20, 45].

Our subgroup analysis revealed variations in the incidence of adverse outcomes across different sarcopenia definition groups, likely due to differences in cutoff values that affect prevalence assessment [10, 47]. A previous study similarly demonstrated that baseline sarcopenia was associated with increased mortality risk, with differences in predictive ability across definitions, consistent with our results [48]. In this study, the applied criteria varied in both thresholds and emphasis on muscle function versus mass. Definitions incorporating functional measures such as handgrip strength and gait speed may better capture clinically relevant frailty, whereas mass-only criteria may underestimate risk. Although several standardized definitions have been proposed [6, 8, 49, 50], some studies have not strictly adhered to them. This limits comparability and underscores the need for future research to adopt consistent definitions.

In addition to diagnostic differences, methods for assessing muscle mass also vary across studies. In this study, four different assessment methods were employed. CT provides highly accurate and precise measurements of muscle mass and composition, but it is costly and not always accessible for routine use. Most of the current studies use DXA and BIA to measure muscle mass [8]. DXA offers good accuracy and is more accessible than CT, but still requires specialized equipment [51]. BIA is quick and noninvasive, but the results can be influenced by hydration status and are less reliable in certain populations [51].

We found that adjusted data from several studies with a low risk of methodological bias showed that sarcopenic T2DM patients had an increased mortality risk compared with nonsarcopenic patients. These findings indicate that sarcopenia is a predictor of adverse mortality in this population. Sarcopenia significantly elevates the risk of mortality, osteoporotic fractures, and cognitive decline [52, 53]. The association between sarcopenia and mortality has been confirmed in many studies across various settings and subpopulations [54–56]. Consistent with these results, the present study revealed that sarcopenia is a risk factor and has a significant negative effect on survival in T2DM patients. Substantial evidence suggests that patients with sarcopenia experience higher rates of adverse outcomes, including CVD and postoperative complications [57, 58]. Although our findings were consistent across studies, most data came from Asian cohorts, which limits their generalizability. Ethnic differences in body composition, lifestyle, and genetics may influence both the prevalence and prognostic impact of sarcopenia. Future studies in multi-ethnic populations are needed to validate our findings.

The association between sarcopenia and the development of cardiovascular disease aligns with the growing recognition of sarcopenia as a cardiovascular risk factor. The increased HRs for sarcopenia and CVD emphasize the need for integrated cardiovascular care in individuals with sarcopenia [59]. Moreover, the association with presarcopenia suggests that prompt intervention may mitigate cardiovascular risk.

Our findings revealed that, compared with those without sarcopenia, sarcopenic T2DM patients present with increased diabetes-associated complications. Among the included studies, four reported diabetes-associated renal dysfunction and other complications, including CVD, diabetes-induced dementia, and diabetic stroke. Although we pooled the adjusted HRs and ORs for different complications, the heterogeneity statistics (I^2^ = 0.0%; I^2^ = 35%) suggested that these studies had no significant heterogeneity. T2DM patients with sarcopenia have a greater risk of diabetes-associated complications, indicating that sarcopenia may be a predictor of adverse outcomes in this population.

Some limitations in our study should be acknowledged. First, significant heterogeneity existed in the diagnostic criteria for sarcopenia across the included studies. Notably, many of the studies assessed sarcopenia based on muscle mass alone, without strictly adhering to the current consensus guidelines. This methodological inconsistency may compromise diagnostic accuracy and potentially weaken observed associations with clinical outcomes, as mass-only definitions may not fully capture the functional impairment central to sarcopenia. Second, the geographic distribution of studies limits generalizability, with most studies conducted in Asian populations. Extrapolation to non-Asian ethnic groups remains uncertain, given potential variations in body composition, muscle mass thresholds, lifestyle factors, and genetic predisposition that may influence sarcopenia’s clinical implications in T2DM. Third, all included studies were observational studies. While these investigations provide valuable evidence for associations between sarcopenia and adverse outcomes in T2DM, they cannot establish causality. Fourth, the quantitative synthesis was constrained by the relatively small number of eligible studies available for meta-analysis, particularly in subgroup analyses, where several parameters contained fewer studies. Fifth, our inclusion criteria restricted the selection to English-language publications, potentially introducing language bias and excluding relevant data from non-English sources. Furthermore, funnel plot asymmetry indicated possible publication bias favoring significant positive findings, though we employed trim-and-fill adjustment where appropriate. Although we implemented comprehensive search strategies and statistical adjustments, these limitations suggest that our findings should be interpreted with caution.

## Conclusion

In conclusion, our analysis supported sarcopenia as a predictor of increased all-cause mortality and complications in T2DM patients. These findings underscore the clinical imperative for integrating sarcopenia assessment into routine T2DM management to facilitate early risk stratification. Consequently, future research should prioritize developing and validating standardized screening protocols in T2DM clinics.

### Certainty of evidence

The GRADE system was used to evaluate the certainty of evidence. The evidence for all-cause mortality was rated very low due to suspected publication bias. Evidence for CVD risk and diabetic complications measured by HR was rated low, whereas evidence for diabetic complications measured by OR was rated moderate due to a strong association(see Supplementary Table 6).

### Publication bias and sensitivity analysis

Funnel plot symmetry revealed evidence of publication bias among studies for mortality. In contrast, no significant evidence of publication bias was observed for CVD or complications (see Supplementary Fig. 7). Begg’s test did not indicate publication bias (mortality: *p* = 0.452; CVD: *p* = 0.220; complications: *p* = 0.308). However, Egger’s test indicated small-study effects for mortality (*p* = 0.012), while no significant bias was detected for CVD (*p* = 0.163) or complications (*p* = 0.099) (see Supplementary Fig. 8). Overall, these findings suggest potential publication bias for mortality but not for CVD or complications (see Supplementary Table 3). Furthermore, the trim-and-fill analysis indicated that publication bias did not materially change the overall mortality (HR = 1.43, 95% CI = 1.20–1.71), CVD (HR = 1.90, 95% CI = 1.65–2.20), or complication (HR = 1.12, 95% CI = 1.09–1.15) results, shown in Supplementary Fig. 9. In addition, Sensitivity analysis showed no significant differences, indicating that the results of this meta-analysis were robust and not materially affected by the omission of any single study, as shown in Supplementary Fig. 10.

## Supplementary Information

Supplementary Material 1.

## Data Availability

The datasets used and analyzed during the current study are available from the corresponding author upon reasonable request.

## Abbreviations

AWGS: Asian working group on sarcopenia
BIA: Bioelectrical impedance analysis
CT: Computed tomography
CVD: Cardiovascular disease
CKD: Chronic kidney disease
CSS: Cross-sectional study
CI: Confidence intervals
DXA: Dual-energy X-ray absorptiometry
EWGSOP: European working group on sarcopenia in older people
FNIH: Foundation for the national institutes of health
HR: Hazard ratios
NA: Not available
NR: Not reported
OR: Odds ratios
PCS: Prospective cohort study
RCS: Retrospective cohort study
SMI: Skeletal muscle mass index
T2DM: Type 2 diabetes mellitus
