# Supplementary material for: Sarcopenia as a predictor of negative health outcomes in patients with type 2 diabetes mellitus: a systematic review and meta-analysis
Source: Diabetol Metab Syndr. 2025 Nov 5;17:416. doi: 10.1186/s13098-025-01998-w (PMC12590590; doi:10.1186/s13098-025-01998-w)
Supplement: Supplementary file 1 — Supplementary Material 1. [file 13098_2025_1998_MOESM1_ESM.zip › Supplementary Materails/Supplementary Table 1.docx]

| Database:PubMed:2025.3.12 | 1296records |
| --- | --- |
| #1 ("Diabetes Mellitus, Type 2"[MeSH Terms] OR "Diabetes Mellitus, Type 2"[Title/Abstract] OR "Diabetes Mellitus, Type II"[Title/Abstract] OR "Type 2 Diabetes Mellitus"[Title/Abstract] OR "Type 2 Diabetes"[Title/Abstract] OR Diabetes, Type 2[Title/Abstract]) | 257049 |
| #2 "Sarcopenia"[Mesh] OR Sarcopenia[Title/Abstract] OR "muscle wasting"[Title/Abstract] OR "muscle atrophy"[Title/Abstract] OR "muscle mass"[Title/Abstract] OR "muscle strength"[Title/Abstract] | 85206 |
| #3 (((Review[Publication Type]) OR (Letter[Publication Type])) OR (Editorial[Publication Type])) OR (Comment[Publication Type]) | 5887788 |
| #4 #1 AND #2 NOT #3 | 1296 |
|  |  |
| Database:Web Of Science | 2348 records |
| #1 TS=("Diabetes Mellitus, Type 2" OR "Diabetes Mellitus, Type II" OR "Type 2 Diabetes Mellitus"OR "Type 2 Diabetes" OR " Diabetes, Type 2") | 358387 |
| #2 TS=(Sarcopenia OR "muscle wasting" OR "muscle atrophy" OR "muscle mass" OR "muscle strength") | 143992 |
| #3 TS=(“Review” OR “Review of Literature” OR “Systematic Review” OR “Meta-Analysis” OR “Meta Analysis” OR “Network Meta-Analysis” OR “Network Meta Analysis”) | 3801356 |
| #4 #1 AND #2 NOT #3 | 2348 |
|  |  |
| Database:Scoups | 2094 records |
| #1 TITLE-ABS-KEY("Diabetes Mellitus, Type 2" OR "Diabetes Mellitus, Type II" OR "Type 2 Diabetes Mellitus"OR "Type 2 Diabetes" OR " Diabetes, Type 2") | 279515 |
| #2 TITLE-ABS-KEY(Sarcopenia OR "muscle wasting" OR "muscle atrophy" OR "muscle mass" OR "muscle strength") | 189509 |
| #3 TITLE-ABS-KEY(“Review” OR “Review of Literature” OR “Systematic Review” OR “Meta-Analysis” OR “Meta Analysis” OR “Network Meta-Analysis” OR “Network Meta Analysis”) | 6795063 |
| #4 #1 AND #2 AND NOT #3 | 2094 |
|  |  |
| Database:Embase | 1408 records |
| #1 'type 2 diabet*':ab,ti OR 'non insulin dependent diabetes mellitus'/exp OR 'non insulin dependent diabetes mellitus':ab,ti OR 'non insulin dependent diabet*':ab,ti OR 'type2 diabet*':ab,ti OR 'type ii diabet*':ab,ti OR 'typeii diabet*':ab,ti | 456718 |
| #2 Sarcopenia/exp OR Sarcopenia:ab,ti OR ‘muscle wasting’:ab,ti OR ‘muscle atrophy’:ab,ti OR ‘muscle mass’:ab,ti OR ‘muscle strength’:ab,ti | 119226 |
| #3 (“Review” OR “Review of Literature” OR “Systematic Review” OR “Meta-Analysis” OR “Meta Analysis” OR “Network Meta-Analysis” OR “Network Meta Analysis”):ab,ti | 3140361 |
| #4 #1 AND #2 NOT #3 | 1408 |
