# Supplementary material for: Sarcopenia as a predictor of negative health outcomes in patients with type 2 diabetes mellitus: a systematic review and meta-analysis
Source: Diabetol Metab Syndr. 2025 Nov 5;17:416. doi: 10.1186/s13098-025-01998-w (PMC12590590; doi:10.1186/s13098-025-01998-w)
Supplement: Supplementary file 1 — Supplementary Material 1. [file 13098_2025_1998_MOESM1_ESM.zip › Supplementary Materails/Supplementary Table 2.docx]

Supplementary Table Study Quality for cohort study

| **Study** | **Representativeness of the exposed cohort** | **Selection of the nonexposed cohort** | **Ascertainment of exposure** | **Demonstration of outcome of interest** | **Basis of the design or analysis** | **Assessment of outcome** | **Appropriate follow‑up length for outcomes** | **Adequate follow‑up** | **Total Score** |
| --- | --- | --- | --- | --- | --- | --- | --- | --- | --- |
| **Wei** | ★ | ★ | ★ | ★ | ★★ | ★ | ★ | ★ | 9 |
| **Boonpor** | ★ | ★ | ★ | ★ | ★★ | ★ | ★ | ★ | 9 |
| **Beretta** | ★ | ★ | ★ |  | ★ | ★ | ★ | ★ | 7 |
| **Sun** | ★ | ★ | ★ | ★ | ★★ | ★ |  |  | 7 |
| **Ye** | ★ | ★ | ★ | ★ | ★ | ★ | ★ | ★ | 8 |
| **Lin** | ★ | ★ | ★ | ★ | ★ | ★ | ★ | ★ | 8 |
| **Chuan** | ★ | ★ | ★ | ★ | ★★ | ★ | ★ | ★ | 9 |
| **Huang** | ★ | ★ | ★ | ★ | ★ | ★ | ★ | ★ | 8 |
| **Chai** | ★ | ★ | ★ | ★ | ★★ | ★ | ★ | ★ | 9 |
| **Takahashi** | ★ | ★ | ★ | ★ | ★ |  | ★ | ★ | 7 |
| **Fukuda** | ★ | ★ | ★ | ★ | ★★ | ★ | ★ | ★ | 9 |
| **Bouchi** | ★ | ★ | ★ | ★ | ★ | ★ | ★ | ★ | 8 |

Supplementary Table Study Quality for cross-sectional study

| **Study** | **1** | **2** | **3** | **4** | **5** | **6** | **7** | **Total Score** |
| --- | --- | --- | --- | --- | --- | --- | --- | --- |
| **Park** | YES:✔  NO:  Unclear:  NA: | YES:✔  NO:  Unclear:  NA: | YES:✔  NO:  Unclear:  NA: | YES:✔  NO:  Unclear:  NA: | YES:✔  NO:  Unclear:  NA: | YES:✔  NO:  Unclear:  NA: | YES:✔  NO:  Unclear:  NA: | 7 |
| **Chung** | YES:✔  NO:  Unclear:  NA: | YES:✔  NO:  Unclear:  NA: | YES:✔  NO:  Unclear:  NA: | YES:✔  NO:  Unclear:  NA: | YES:✔  NO:  Unclear:  NA: | YES:✔  NO:  Unclear:  NA: | YES:✔  NO:  Unclear:  NA: | 7 |
| **Yang** | YES:✔  NO:  Unclear:  NA: | YES:✔  NO:  Unclear:  NA: | YES:✔  NO:  Unclear:  NA: | YES:✔  NO:  Unclear:  NA: | YES:✔  NO:  Unclear:  NA: | YES:✔  NO:  Unclear:  NA: | YES:✔  NO:  Unclear:  NA: | 7 |

1.Were the criteria for inclusion in the sample clearly defined?

2. Were the study subjects and the setting described in detail?

3. Were objective, standard criteria used for measurement of the condition?

4. Were confounding factors identified?

5. Were strategies to deal with confounding factors stated?

6. Were the outcomes measured in a valid and reliable way?

7.Was appropriate statistical analysis used?
