# Supplementary material for: Sarcopenia as a predictor of negative health outcomes in patients with type 2 diabetes mellitus: a systematic review and meta-analysis
Source: Diabetol Metab Syndr. 2025 Nov 5;17:416. doi: 10.1186/s13098-025-01998-w (PMC12590590; doi:10.1186/s13098-025-01998-w)
Supplement: Supplementary file 1 — Supplementary Material 1. [file 13098_2025_1998_MOESM1_ESM.zip › Supplementary Materails/Supplementary Table 5.docx]

**Supplementary Table S4. Studies excluded (n=47) with reasons**

| Studies excluded | Reasons |
| --- | --- |
| Alfaro-Alvarado 2023[1] | Irrelevant outcomes |
| Beretta 2018[2] | Ineligible population |
| Bulur 2023[3] | Irrelevant outcomes |
| Chen 2024[4] | Irrelevant outcomes |
| Cheng 2017[5] | Irrelevant outcomes |
| Choi 2014[6] | Irrelevant outcomes |
| Choi 2013[7] | Conference abstract |
| Deng 2024[8] | Irrelevant outcomes |
| Hamasaki 2017[9] | Irrelevant disease focus |
| Harrabi 2018[10] | No available data |
| Hashimoto 2016[11] | Irrelevant outcomes |
| Hayón-Ponce 2021[12] | Non-English literature |
| Ida 2019[13] | Irrelevant outcomes |
| Ida 2019[14] | Ineligible population |
| Ida 2018[15] | Ineligible population |
| Imaizumi 2016[16] | Irrelevant outcomes |
| Jiang 2021[17] | Non-English literature |
| Lin 2022[18] | Irrelevant disease focus |
| Low 2022[19] | Irrelevant outcomes |
| Miyake 2017[20] | Conference abstract |
| Miyake 2019[21] | Irrelevant disease focus |
| Morikawa 2021[22] | Irrelevant disease focus |
| Nakanishi 2020[23] | Irrelevant outcomes |
| Osaka 2023[24] | Conference abstract |
| Seo 2020[25] | Irrelevant outcomes |
| Seo 2020[26] | Irrelevant outcomes |
| Shin 2016[27] | Conference abstract |
| Shin 2017[28] | Irrelevant outcomes |
| Shin 2023[29] | Irrelevant outcomes |
| Sung 2020[30] | Irrelevant outcomes |
| Xia 2024[31] | No available data |
| Xia 2023[32] | Irrelevant outcomes |
| Xu 2024[33] | Irrelevant outcomes |
| Xu 2024[34] | Irrelevant outcomes |
| Yan 2020[35] | No available data |
| Zhang 2020[36] | Irrelevant outcomes |
| Zhang 2022[37] | Irrelevant outcomes |
| Zhang 2020[38] | Irrelevant outcomes |
| Mamatha 2020[39] | Irrelevant disease focus |
| Khan 2017[40] | Irrelevant outcomes |
| Kaji 2019[41] | Irrelevant outcomes |
| Jung 2020[42] | Irrelevant outcomes |
| Jung 2018[43] | Irrelevant disease focus |
| Çeliker 2018[44] | Irrelevant outcomes |
| Beretta 2023[45] | Ineligible population |
| Fukuda 2017[46] | Irrelevant outcomes |
| Belfield 2024[47] | Irrelevant outcomes |

1. Alfaro-Alvarado FA, Rosas-Barrientos JV, Ocharan-Hernandez ME, Diaz-Chiguer D, Vargas-De-Leon C. Association between Sarcopenia and Poor Glycemic Control in Older Adults with Type 2 Diabetes Mellitus. *Dis Basel Switz*. 2023;11(4). doi:10.3390/diseases11040175

2. Beretta MV, Feldman J, Nery C, Da Costa Rodrigues T. Sarcopenia and the not presence of diabetes as a predictor of mortality after hospital discharge in elderly patients. *Diabetol Metab Syndr*. 2018;10. doi:10.1186/s13098-018-0315-8

3. Bulur A, Sivritepe R. The Association between Non-Alcoholic Fatty Liver Disease and Dynapenia in Men Diagnosed with Type 2 Diabetes Mellitus. *Healthc Switz*. 2023;11(2). doi:10.3390/healthcare11020243

4. Chen Y, Liao J, Zeng Y, et al. The combined effect of diabetes mellitus and sarcopenia on depression and cognitive function: insights from the CHARLS cohort, 2011–2020. *Eur Geriatr Med*. 2024;15(6):1881-1890. doi:10.1007/s41999-024-01039-1

5. Cheng Q, Hu J, Yang P, et al. Sarcopenia is independently associated with diabetic foot disease. *Sci Rep*. 2017;7(1):8372. doi:10.1038/s41598-017-08972-1

6. Choi YJ, Kim SK, Kwak JJ, Park SW, Lee EJ, Huh KB. Age-related skeletal muscle loss as an independent predictor of NAFLD risk in Korean women with type 2 diabetes. *Diabetes Res Clin Pr*. 2014;106:S162-S163.

7. Choi YJ, Lim JS, Jee SH, et al. Low relative skeletal muscle mass is independently associated with NAFLD in Korean women with type 2 diabetes. *Diabetologia*. 2013;56:S527. doi:10.1007/s00125-013-3012-z

8. Deng S, Lv S, Liu Y, et al. Low Muscle Mass is Independently Associated with an Increased Risk of Having Lower Limb Atherosclerosis in T2DM Patients. *Diabetes Metab Syndr Obes*. 2024;17:4211-4221. doi:10.2147/DMSO.S492973

9. Hamasaki H, Kawashima Y, Katsuyama H, Sako A, Goto A, Yanai H. Association of handgrip strength with hospitalization, cardiovascular events, and mortality in Japanese patients with type 2 diabetes. *Sci Rep*. 2017;7(1):7041. doi:10.1038/s41598-017-07438-8

10. Harrabi T, Zahra H, Khiari M, et al. Sarcopenia and diabetes: The longevity of our muscles is at risk. *Osteoporos Int*. 2018;29(1):S530. doi:10.1007/s00198-018-4465-1

11. Hashimoto Y, Osaka T, Fukuda T, Tanaka M, Yamazaki M, Fukui M. The relationship between hepatic steatosis and skeletal muscle mass index in men with type 2 diabetes. *Endocr J*. 2016;63(10):877-884. doi:10.1507/endocrj.EJ16-0124

12. Hayón-Ponce M, García-Fontana B, Avilés-Pérez M, González-Salvatierrra S, Moratalla-Aranda E, Muñoz-Torres B. Fuerza muscular como predictora de fragilidad ósea en pacientes con diabetes mellitus tipo 2 X1 Muscle strength as a predictor of bone fragility in patients with type 2 diabetes mellitus. *Rev Osteoporos Metab Miner*. 2021;13(4):137-144. doi:10.4321/s1889-836x2021000400006

13. Ida S, Kaneko R, Nagata H, et al. Association between Sarcopenia and Overactive Bladder in Elderly Diabetic Patients. *J Nutr Health AGING*. 2019;23(6):532-537. doi:10.1007/s12603-019-1190-1

14. Ida S, Kaneko R, Nagata H, et al. Association between sarcopenia and sleep disorder in older patients with diabetes. *Geriatr Gerontol Int*. 2019;19(5):399-403. doi:10.1111/ggi.13627

15. Ida S, Murata K, Nakai M, et al. Relationship between sarcopenia and depression in older patients with diabetes: An investigation using the Japanese version of SARC-F. *Geriatr Gerontol Int*. 2018;18(9):1318-1322. doi:10.1111/ggi.13461

16. Imaizumi Y, Eguchi K, Yamamoto M, Kaku R, Kario K. Sarcopenia is independently associated with arterial stiffness in patients with type 2 diabetes mellitus. *Circulation*. 2016;134. http://www.embase.j.sjuku.top/search/results?subaction=viewrecord&id=L619218907&from=export U2 - L619218907

17. Jiang H, Cheng QF, Zhu BZ, Yang Q. Study on the relationship between non-alcoholic fatty liver disease and sarcopenia in type 2 diabetes mellitus. *Zhonghua Gan Zang Bing Za Zhi Zhonghua Ganzangbing Zazhi Chin J Hepatol*. 2021;29(10):977-982. doi:10.3760/cma.j.cn501113-20201018-00560

18. Lin X, Chen Z, Huang H, Zhong J, Xu L. Diabetic kidney disease progression is associated with decrease

lower-limb muscle mass and increased visceral fat area in T2DM patients. *Front Endocrinol*. 2022;13. doi:10.3389/fendo.2022.1002118

19. Low S, Goh KS, Ng TP, et al. Decline in skeletal muscle mass is associated with cognitive decline in type 2 diabetes mellitus. *J Diabetes Complications*. 2022;36(9):108258. doi:10.1016/j.jdiacomp.2022.108258

20. Miyake H, Kanazawa I, Sugimoto T. Low skeletal muscle mass is at increased risk of all-cause mortality in patients with type 2 diabetes. *Diabetologia*. 2017;60(1):S556-S557. doi:10.1007/s00125-017-4350-z

21. Miyake H, Kanazawa I, Tanaka K ichiro, Sugimoto T. Low skeletal muscle mass is associated with the risk of all-cause mortality in patients with type 2 diabetes mellitus. *Ther Adv Endocrinol Metab*. 2019;10. doi:10.1177/2042018819842971

22. Morikawa Y, Kawakami R, Horii M, Yamamoto Y, Yabuta M, Saito Y. Handgrip strength is an independent predictor of cardiovascular outcomes in diabetes mellitus. *Int Heart J*. 2021;62(1):50-56. doi:10.1536/ihj.20-677

23. Nakanishi S, Iwamoto M, Shinohara H, Iwamoto H, Kaneto H. Impact of sarcopenia on glycemic control and atherosclerosis in Japanese patients with type 2 diabetes: Cross-sectional study using outpatient clinical data. *Geriatr Gerontol Int*. 2020;20(12):1196-1201. doi:10.1111/ggi.14063

24. Osaka T, Fukui M. IDF2022-0754 Sarcopenia is a risk factor for progression of diabetic nephropathy in type 2 diabetes. *Diabetes Res Clin Pr*. 2023;197. doi:10.1016/j.diabres.2023.110390

25. Seo DH, Lee YH, Park SW, et al. Sarcopenia is associated with non-alcoholic fatty liver disease in men with type 2 diabetes. *Diabetes Metab*. 2020;46(5):362-369. doi:10.1016/j.diabet.2019.10.004

26. Seo DH, Lee YH, Suh YJ, et al. Low muscle mass is associated with carotid atherosclerosis in patients with type 2 diabetes. *Atherosclerosis*. 2020;305:19-25. doi:10.1016/j.atherosclerosis.2020.05.021

27. Shin JY. Association between renal dysfunction and sarcopenia in Korean patients with type 2 diabetes. *Diabetes*. 2016;65:A382. doi:10.2337/db16-1375-1656

28. Shin JY. Association of higher free thyroxine levels with sarcopenia in Korean patients with type 2 diabetes. *Diabetes*. 2017;66:A400.

29. Shin JY. Appendicular Skeletal Muscle Mass and Grip Strength in Relation to the Progression of Carotid Plaque Score in Patients with Type 2 Diabetes. *Diabetes*. 2023;72. doi:10.2337/db23-466-P

30. Sung MJ, Lim TS, Jeon MY, et al. Sarcopenia Is Independently Associated with the Degree of Liver Fibrosis in Patients with Type 2 Diabetes Mellitus. *GUT LIVER*. 2020;14(5):626-635. doi:10.5009/gnl19126

31. Xia LF, Li JB, Tian GS, et al. Effect of Sarcopenia on 10-Year Risk of Atherosclerotic Cardiovascular Disease in Patients with Type 2 Diabetes Mellitus. *Diabetes Metab Syndr Obes*. 2024;17:1621-1634. doi:10.2147/DMSO.S450225

32. Xia N, Jiahui C, Qiuhong H, Zhipeng D, Ziwei T, Qingfeng C. Relationship between sarcopenia and polyvascular disease in diabetic foot patients. *Chin J Diabetes Mellit*. 2023;15(1):26-31. doi:10.3760/cma.j.cn115791-20220613-00271

33. Xu H, Xiang QY, Zhan JK, et al. Association between macro- and microvascular damage and sarcopenia index in individuals with type 2 diabetes mellitus. *Appl Physiol Nutr Metab*. 2024;49(6):762-772. doi:10.1139/apnm-2023-0476

34. Xu K, Feng X, Xu Z, Pan Y, Zhang P, Zhu H. Association of sarcopenia with osteoporosis in Chinese patients with type 2 diabetes. *BMC Musculoskelet Disord*. 2024;25(1). doi:10.1186/s12891-024-07323-2

35. Yan J, Zheng K, Lin C, Liu C. Correlation between sarcopenia and albuminuria in patients with type 2 diabetes. *Nan Fang Yi Ke Da Xue Xue Bao*. 2020;40(3):407-412. doi:10.12122/j.issn.1673-4254.2020.03.21

36. Zhang Y, Ren L, Zheng F, et al. Correlation between lower extremity arterial disease and skeletal muscle mass in patients with type 2 diabetes mellitus. *J Int Med Res*. 2020;48(3). doi:10.1177/0300060519897483

37. Zhang Y, Weng S, Huang L, Shen X, Zhao F, Yan S. Association of sarcopenia with a higher risk of infection in patients with type 2 diabetes. *Diabetes Metab Res Rev*. 2022;38(1). doi:10.1002/dmrr.3478

38. Zhang Y, Shen X, He L, Zhao F, Yan S. Association of sarcopenia and muscle mass with both peripheral neuropathy and nerve function in patients with type 2 diabetes. *DIABETES Res Clin Pract*. 2020;162. doi:10.1016/j.diabres.2020.108096

39. Mamatha CN, Dhananjaya JR, Sudarshan CR. Association between body mass index and hand grip strength on bone mineral density in type 2 diabetes mellitus – a cross-sectional study. Natl J Physiol Pharm Pharmacol. 2020;10(10):855-860. doi:10.5455/njppp.2020.10.06164202026062020

40. Khan A, Junaid N. Prevalence of diabetic foot syndrome amongst population with type 2 diabetes in Pakistan in primary care settings. J Pak Med Assoc. 2017;67(12):1818-1824.

41. Kaji A, Hashimoto Y, Kobayashi Y, et al. Sarcopenia is associated with tongue pressure in older patients with type 2 diabetes: A cross-sectional study of the KAMOGAWA-DM cohort study. Geriatr Gerontol Int. 2019;19(2):153-158. doi:10.1111/ggi.13577

42. Jung CH, Cho YY, Choi D, Kim BY, Kim CH, Mok JO. Relationship of Sarcopenia with Microcirculation Measured by Skin Perfusion Pressure in Patients with Type 2 Diabetes. Endocrinol Metab. 2020;35(3):578-586. doi:10.3803/EnM.2020.679

43. Jung CH, Cho YY, Choi DH, et al. Low muscle strength rather than low muscle mass is associated with cardiovascular autonomic neuropathy in patients with type 2 diabetes. Sci Rep. 2024;14(1):23385. doi:10.1038/s41598-024-74390-944. Çeliker M, Selçuk MY, Olt S. Sarcopenia in diabetic nephropathy: a cross-sectional study. Romanian J Intern Med Rev Roum Med Interne. 2018;56(2):102-108. doi:10.2478/rjim-2018-000345.

44. Çeliker M, Selçuk MY, Olt S. Sarcopenia in diabetic nephropathy: a cross-sectional study. Romanian J Intern Med Rev Roum Med Interne. 2018;56(2):102-108. doi:10.2478/rjim-2018-0003

45. Beretta MV, Filho FFD, Freiberg RE, Feldman JV, Nery C, Rodrigues TC. Sarcopenia and Type 2 diabetes mellitus as predictors of 2-year mortality after hospital discharge in a cohort of hospitalized older adults. Diabetes Res Clin Pract. 2020;159. doi:10.1016/j.diabres.2019.107969

46. Fukuda T, Bouchi R, Takeuchi T, et al. Association of diabetic retinopathy with both sarcopenia and muscle quality in patients with type 2 diabetes: A crosssectional study. BMJ Open Diabetes Res Care. 2017;5(1):e000404. doi:10.1136/bmjdrc-2017-000404

47. Belfield AE, Wilkinson TJ, Henson J, et al. Sarcopenia prevalence using handgrip strength or chair stand performance in adults living with type 2 diabetes mellitus. Age Ageing. 2024;53(5). doi:10.1093/ageing/afae090
