# Supplementary material for: Sarcopenia as a predictor of negative health outcomes in patients with type 2 diabetes mellitus: a systematic review and meta-analysis
Source: Diabetol Metab Syndr. 2025 Nov 5;17:416. doi: 10.1186/s13098-025-01998-w (PMC12590590; doi:10.1186/s13098-025-01998-w)
Supplement: Supplementary file 1 — Supplementary Material 1. [file 13098_2025_1998_MOESM1_ESM.zip › Supplementary Materails/Supplementary Table 6.docx]

**Supplementary Table 6. GRADE evidence profile for the evidence**

| **Certainty assessment** | | | | | | | | | **Total no.** | **Effect** | | **Certainty** |
| --- | --- | --- | --- | --- | --- | --- | --- | --- | --- | --- | --- | --- |
| **Outcome** | **No. of studies** | **Study design** | **Risk of bias^a^** | **Inconsistency** | **Indirectness** | **Imprecision** | **Publication bias** | **Other considerations** |  | **Relative** | **Absolute** |  |
|  |  |  |  |  |  |  |  |  |  | **(95% CI)** | **(95% CI)** |  |
| Mortality | 6 | non-randomised studies | not serious | not serious | not serious | not serious | undetected | Publication bias suspected^a^ | 216,567 | HR 1.82 (1.28 to 2.32) | 2 fewer per 1,000 (from 2 fewer to 1 fewer) | ⨁◯◯◯ Very Low |
| CVD | 5 | non-randomised studies | not serious | not serious | not serious | not serious | undetected | none | 26,854 | HR 1.94 (1.67 to 2.25) | 2 fewer per 1,000 (from 2 fewer to 1 fewer) | ⨁⨁◯◯ Low |
| Complications(HR) | 4 | non-randomised studies | not serious | not serious | not serious | not serious | undetected | none | 250,818 | HR 1.12 (1.35 to 2.37) | 1 fewer per 1,000 (from 1 fewer to 1 fewer) | ⨁⨁◯◯ Low |
| Complications(OR) | 3 | non-randomised studies | not serious | not serious | not serious | not serious | undetected | Strong association^b^ | 2401 | HR 2.49 (1.53 to 4.05) | 2 fewer per 1,000 (from 4 fewer to 2 fewer) | ⨁⨁⨁◯ Moderate |

CVD,Cardiovascular Disease; CI, confidence interval; HR, hazard ratio; OR,Odds Ratio.

All of the studies we included were observational, hence the Cochrane suggestion is that the grading start at low quality. On GRADE Working Group grades of evidence, **High certainty**: we are very confident that the true effect lies close to that of the estimate of the effect. **Moderate certainty**: we are moderately confident in the effect estimate: the true effect is likely to be close to the estimate of the effect, but there is a possibility that it is substantially different. **Low certainty**: our confidence in the effect estimate is limited: the true effect may be substantially different from the estimate of the effect. **Very low certainty**: we have very little confidence in the effect estimate: the true effect is likely to be substantially different from the estimate of effect.

1. Egger’s test results indicated the possible existence of publication bias.
2. HR:2.49 indicated strong association
