# Supplementary figures and images for: Sarcopenia as a predictor of negative health outcomes in patients with type 2 diabetes mellitus: a systematic review and meta-analysis
Source: Diabetol Metab Syndr. 2025 Nov 5;17:416. doi: 10.1186/s13098-025-01998-w (PMC12590590; doi:10.1186/s13098-025-01998-w)

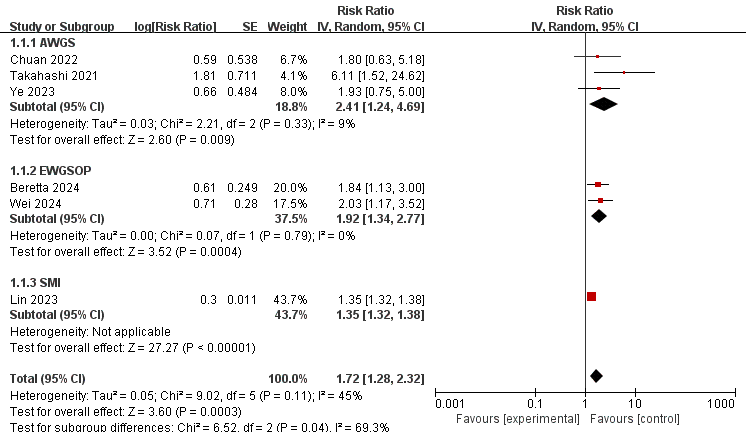

Supplement: Supplementary file 1 — Supplementary Material 1. [file 13098_2025_1998_MOESM1_ESM.zip › Supplementary Materails/Supplementary Figure 1.png]

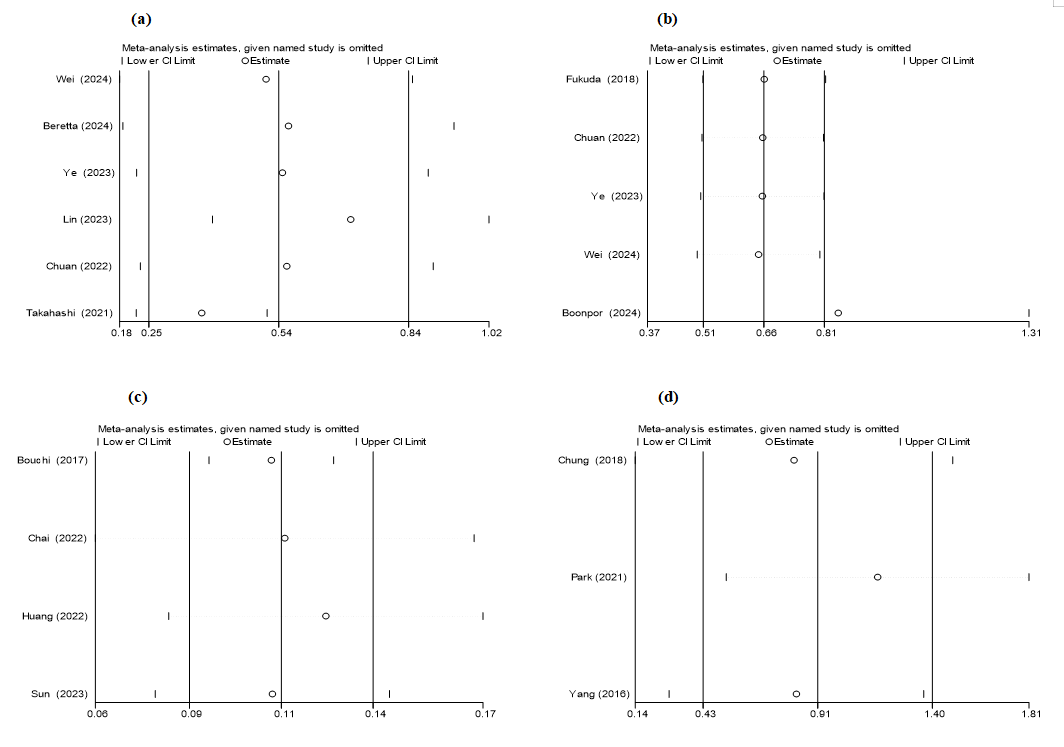

Supplement: Supplementary file 1 — Supplementary Material 1. [file 13098_2025_1998_MOESM1_ESM.zip › Supplementary Materails/Supplementary Figure 10.png]

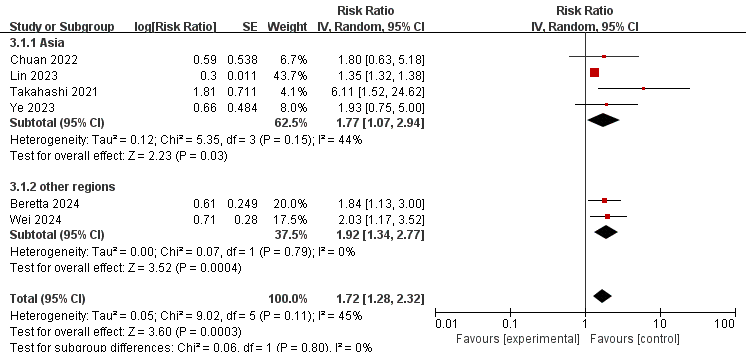

Supplement: Supplementary file 1 — Supplementary Material 1. [file 13098_2025_1998_MOESM1_ESM.zip › Supplementary Materails/Supplementary Figure 2.png]

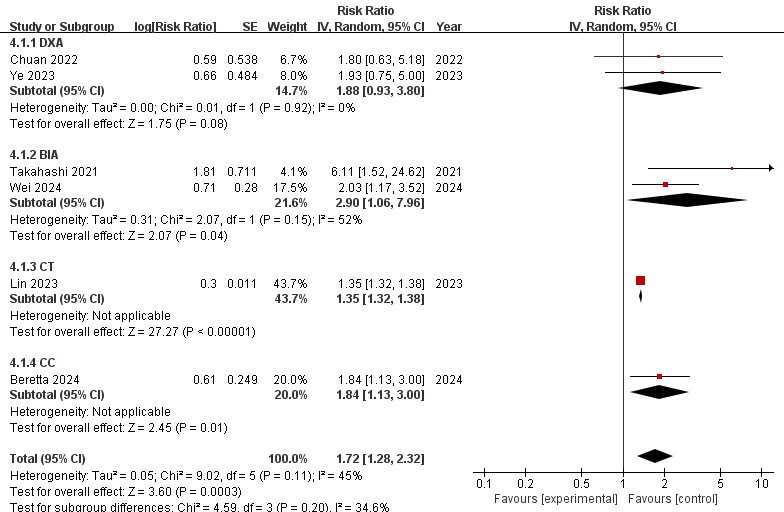

Supplement: Supplementary file 1 — Supplementary Material 1. [file 13098_2025_1998_MOESM1_ESM.zip › Supplementary Materails/Supplementary Figure 3.png]

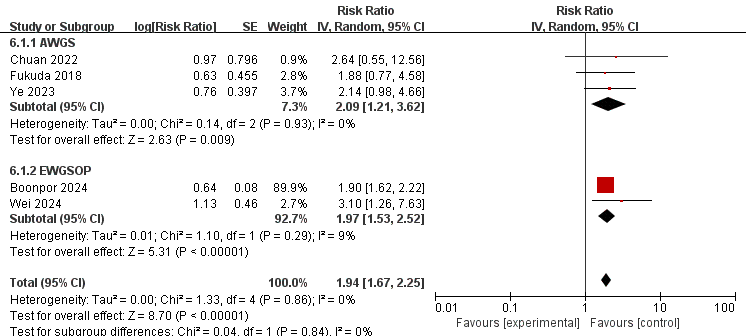

Supplement: Supplementary file 1 — Supplementary Material 1. [file 13098_2025_1998_MOESM1_ESM.zip › Supplementary Materails/Supplementary Figure 4.png]

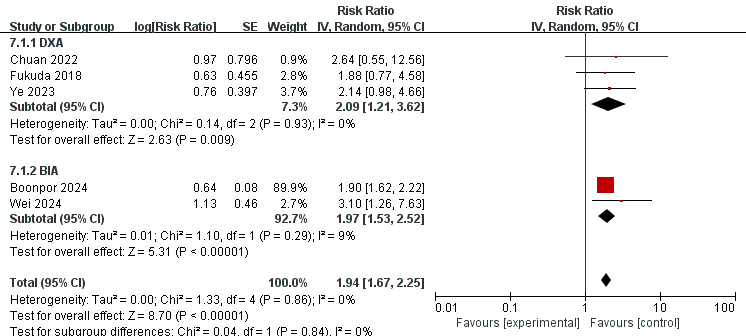

Supplement: Supplementary file 1 — Supplementary Material 1. [file 13098_2025_1998_MOESM1_ESM.zip › Supplementary Materails/Supplementary Figure 5.png]

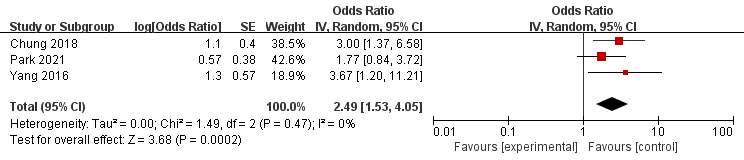

Supplement: Supplementary file 1 — Supplementary Material 1. [file 13098_2025_1998_MOESM1_ESM.zip › Supplementary Materails/Supplementary Figure 6.png]

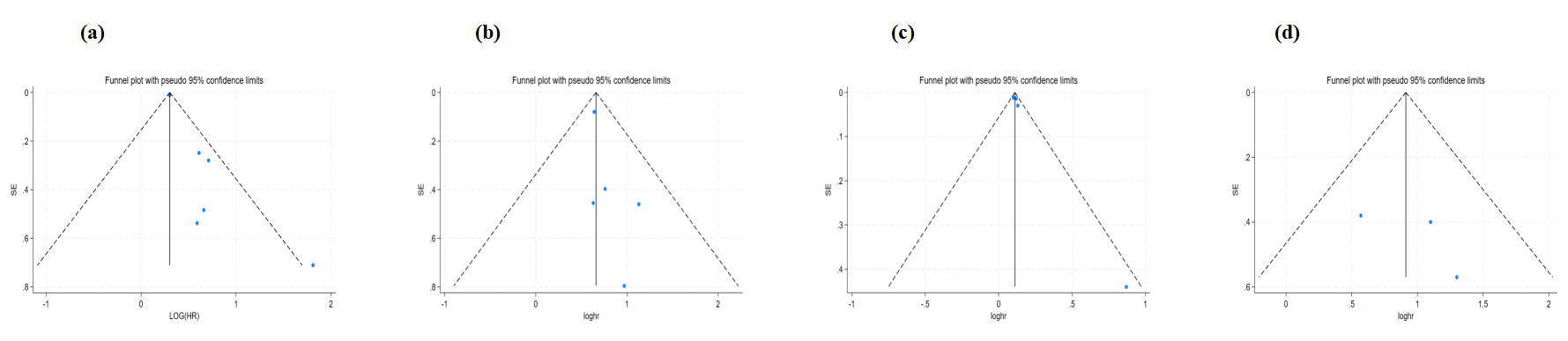

Supplement: Supplementary file 1 — Supplementary Material 1. [file 13098_2025_1998_MOESM1_ESM.zip › Supplementary Materails/Supplementary Figure 7.png]

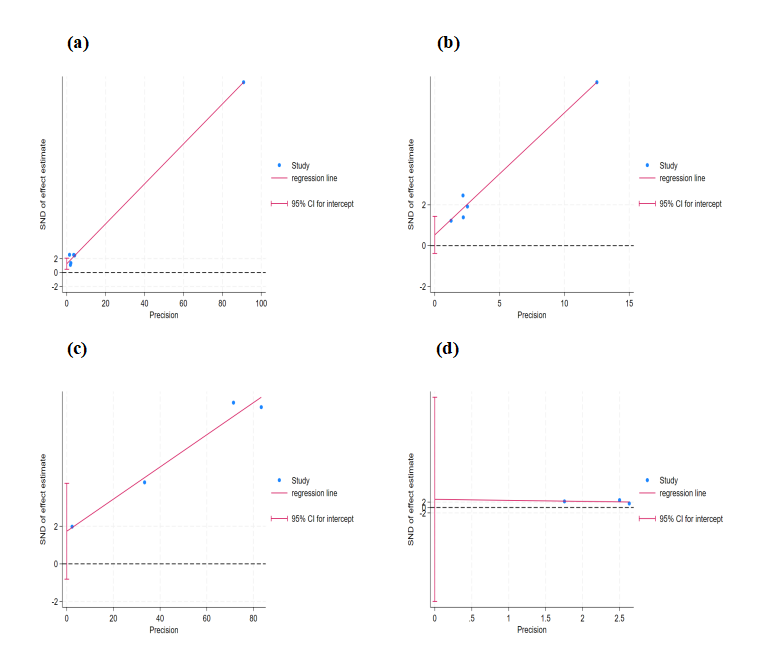

Supplement: Supplementary file 1 — Supplementary Material 1. [file 13098_2025_1998_MOESM1_ESM.zip › Supplementary Materails/Supplementary Figure 8.png]

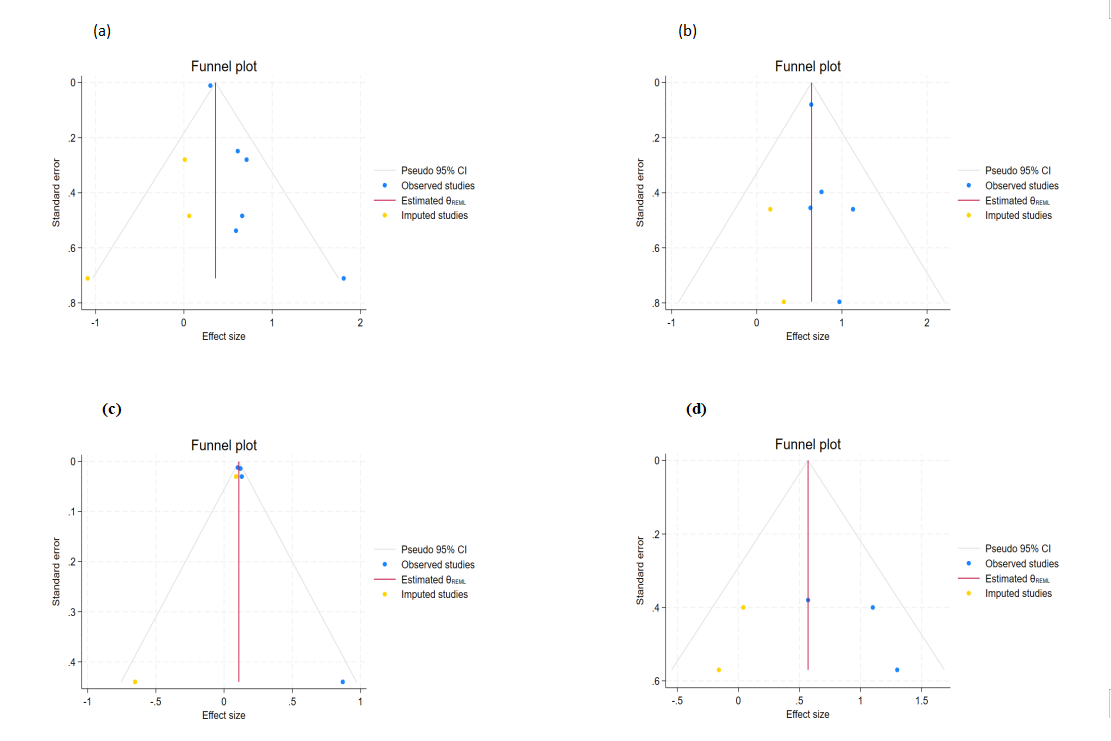

Supplement: Supplementary file 1 — Supplementary Material 1. [file 13098_2025_1998_MOESM1_ESM.zip › Supplementary Materails/Supplementary Figure 9.png]

**Mortality (adjusted HR)**

**
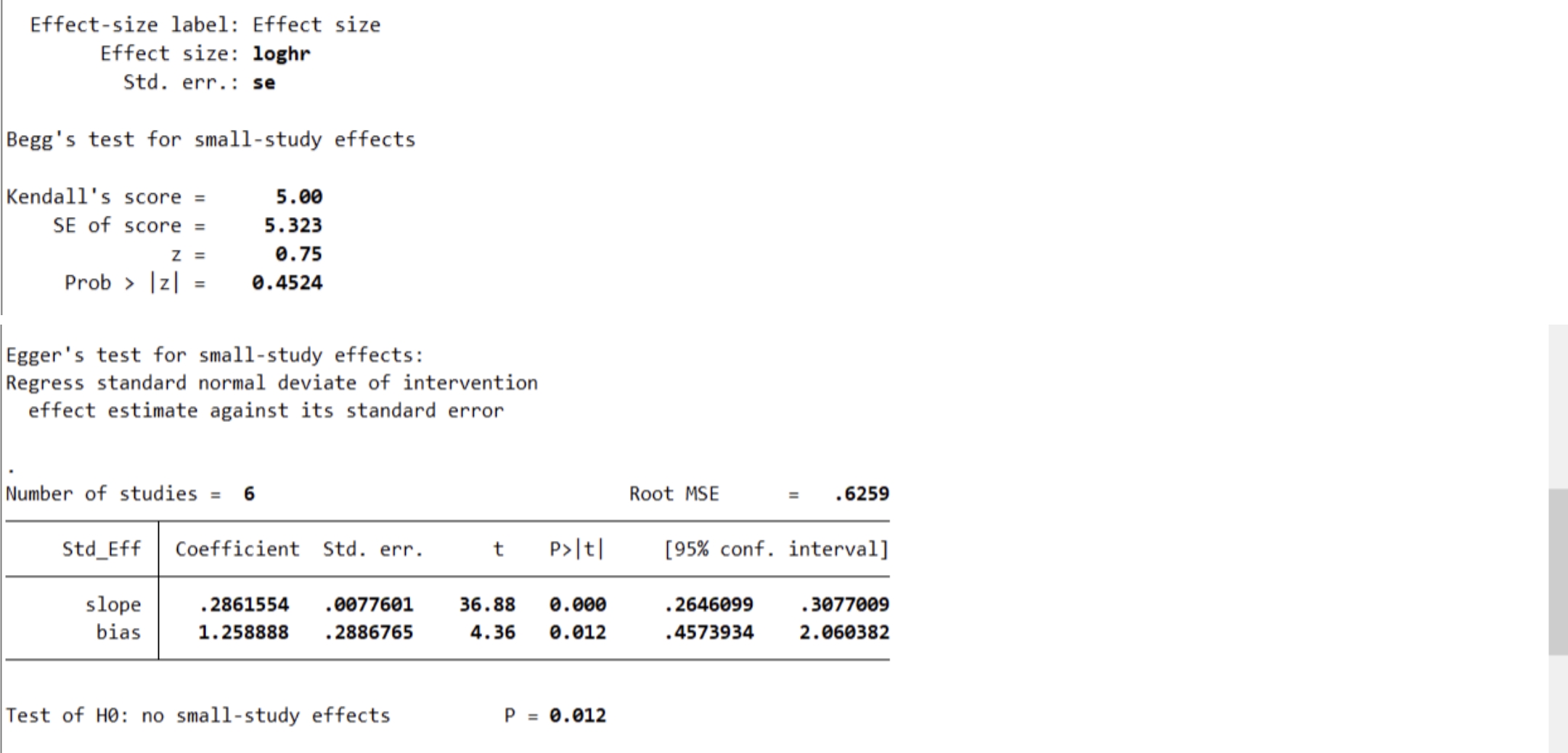
**

**CVD (adjusted HR)**

**
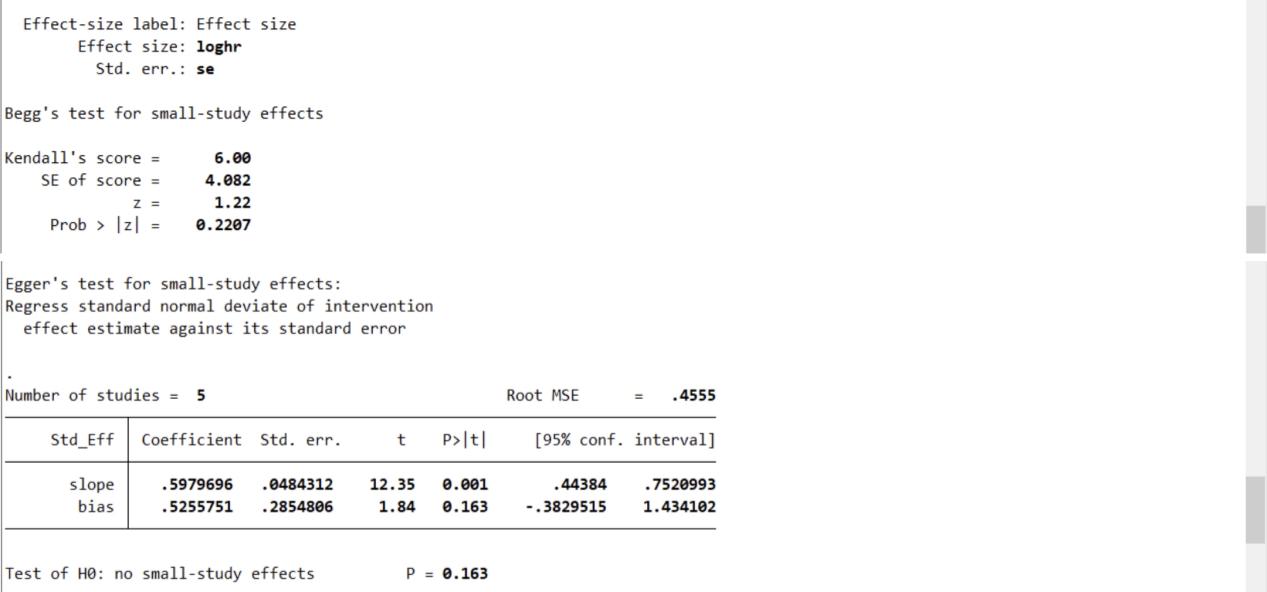
**

**Complications (adjusted HR)**


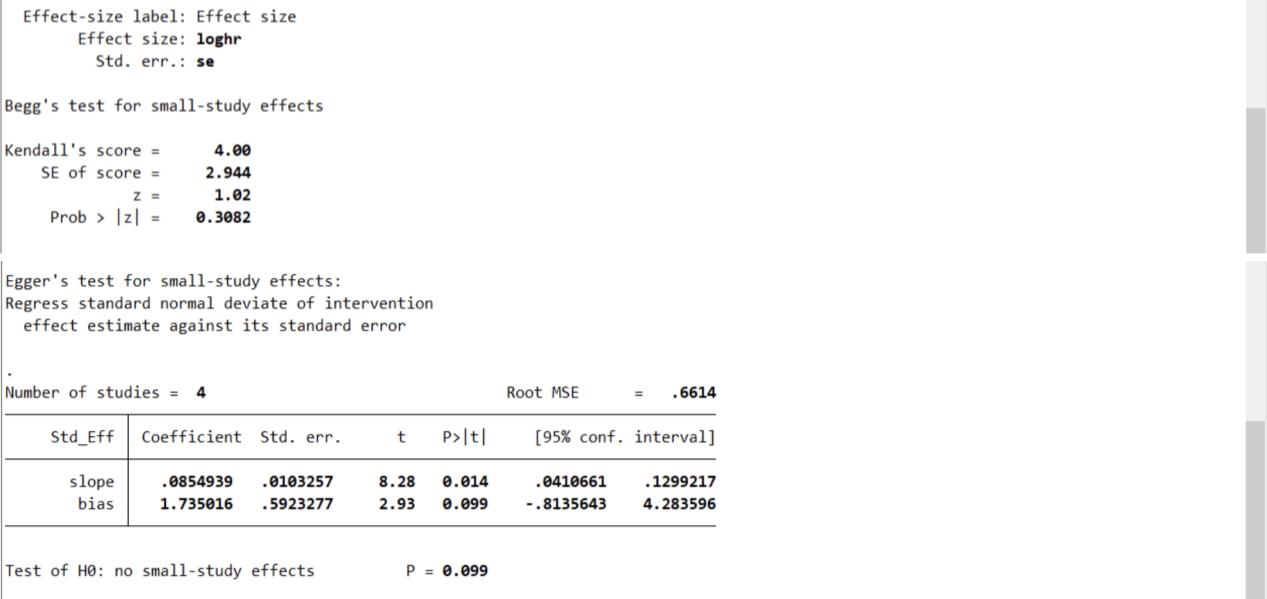


**Complications (adjusted OR)**

**
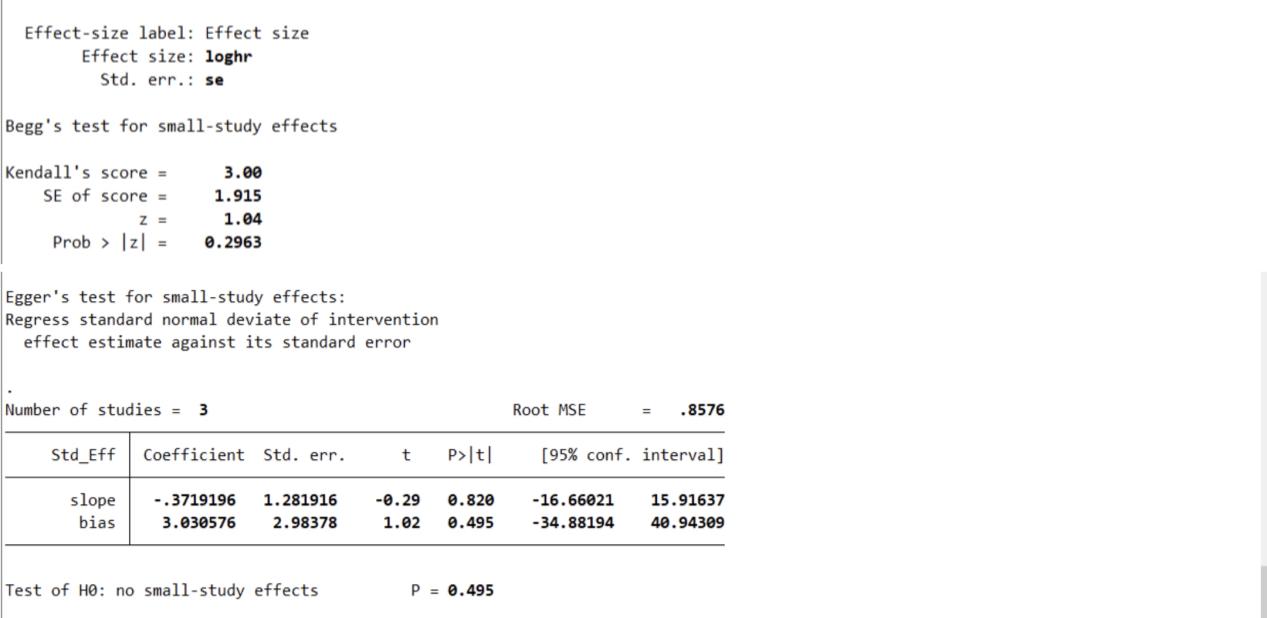
**

Supplement: Supplementary file 1 — Supplementary Material 1. [file 13098_2025_1998_MOESM1_ESM.zip › Supplementary Materails/Supplementary Table 3.docx]
